# Differential surface activation of the A1 domain of von Willebrand factor

**DOI:** 10.1116/1.4943618

**Published:** 2016-03-11

**Authors:** Elaine H. Tronic, Olga Yakovenko, Tobias Weidner, Joe E. Baio, Rebecca Penkala, David G. Castner, Wendy E. Thomas

**Affiliations:** Department of Bioengineering, University of Washington, Seattle, Washington 98195; Department of Chemical Engineering, University of Washington, Seattle, Washington 98195; Department of Bioengineering, University of Washington, Seattle, Washington 98195; Departments of Bioengineering and Chemical Engineering, University of Washington, Seattle, Washington 98195; Department of Bioengineering, University of Washington, Seattle, Washington 98195

## Abstract

The clotting protein von Willebrand factor (VWF) binds to platelet receptor glycoprotein Ibα (GPIbα) when VWF is activated by chemicals, high shear stress, or immobilization onto surfaces. Activation of VWF by surface immobilization is an important problem in the failure of cardiovascular implants, but is poorly understood. Here, the authors investigate whether some or all surfaces can activate VWF at least in part by affecting the orientation or conformation of the immobilized GPIbα-binding A1 domain of VWF. Platelets binding to A1 adsorbed onto polystyrene surfaces translocated rapidly at moderate and high flow, but detached at low flow, while platelets binding to A1 adsorbed onto glass or tissue-culture treated polystyrene surfaces translocated slowly, and detached only at high flow. Both x-ray photoelectron spectroscopy and conformation independent antibodies reported comparable A1 amounts on all surfaces. Time-of-flight secondary ion mass spectrometry (ToF-SIMS) and near-edge x-ray absorption fine structure spectra suggested differences in orientation on the three surfaces, but none that could explain the biological data. Instead, ToF-SIMS data and binding of conformation-dependent antibodies were consistent with the stabilization of an alternative more activated conformation of A1 by tissue culture polystyrene and especially glass. These studies demonstrate that different material surfaces differentially affect the conformation of adsorbed A1 domain and its biological activity. This is important when interpreting or designing *in vitro* experiments with surface-adsorbed A1 domain, and is also of likely relevance for blood-contacting biomaterials.

## I. INTRODUCTION

When a material is placed in a biological environment, the surface of the material acts as the interface between that material and the biological environment.^1^ Proteins attached to a material surface play a critical role in the material/biology interaction and determining the ultimate biological performance of the material.^2^ When biomaterials are placed in contact with blood, plasma proteins attach to these surfaces^2^ and mediate platelet adhesion and activation and thrombosis.^3^ Attempts to prevent adhesion of these proteins to implanted materials such as stents has largely been unsuccessful, as all surfaces appear to bind proteins eventually.^2,3^ Drugs provided orally or eluted from biomaterials can reduce early thrombosis, but also inhibit re-endothelialization, leading to increased risk of late thrombosis.^4^ An alternative approach to regulating platelet activation would be to control the normal healing process. This approach requires understanding the different signals platelets receive from circulating plasma proteins or plasma proteins on injured endothelium, as opposed to plasma proteins immobilized on a material surface. However, the means by which surface immobilization affects the structure and function of such proteins is poorly understood.

Von Willebrand factor (VWF) is a minor component of plasma, but is implicated in acute occlusive episodes in cardiovascular disease, and VWF levels are predictive of frequency and severity of acute events.^5^ Plasma VWF consists of multiple polymerized subunits,^6^ each of which contains an A1 domain that can initiate platelet adhesion by binding to platelet receptor glycoprotein Ibα (GPIbα).^7–9^ VWF circulates in the blood without interacting with platelets unless it is activated by superphysiological levels (80 dyne/cm^2^) of shear stress.^10,11^ However, VWF binds to exposed collagen on injured endothelium and to implanted biomaterials, allowing it to mediate platelet adhesion at shear stresses typical of arterial vasculature (10 dyne/cm^2^).^12^ GPIbα binding to A1 leads to integrin activation and secretion of clotting factors, and thus, formation of a stable clot or thrombus.^13^ As a result, VWF binding to biomaterial implants is a major mediator of thrombotic response and eventual implant failure.

The scientific community remains divided about how VWF is activated. In one model, the A1 domains are masked but become exposed when the large multimers are stretched by flow.^14,15^ In an alternative model, the A1 domain of VWF and the extracellular portion of GPIbα form catch bonds that are longer-lived if exposed to tensile mechanical force,^16^ created when platelets in flow are pulled away from the VWF to which they bind. It remains unclear whether immobilization of VWF onto surfaces activates GPIbα binding by exposing the A1 domain, by stabilizing an alternative conformation of the A1 domain, or by activating catch bonds by anchoring VWF against force. The isolated A1 domain is also commonly used in *in vitro* studies of platelet adhesion,^7,17–19^ typically after immobilization onto a surface. This raises the question as to whether the structure and function of multimeric plasma VWF or the isolated A1 domain is affected by the type of surface to which it is adsorbed.

While methods that are used to determine the atomistic structure of proteins in solution or crystals typically do not provide the sensitivity to examine surface bound proteins, using an approach that combines multiple, complementary surface analysis techniques can provide detailed information about proteins bound to surfaces.^20^ Surface chemistry affects the amount of proteins that bind, as detected by techniques such as antibodies that recognize linear conformation-independent epitopes, x-ray photoelectron spectroscopy (XPS), etc.^21–24^ Surface chemistry can also affect the orientations or conformation of bound proteins,^21,25–30^ which can dictate function. Antibodies that recognize three-dimensional conformation-dependent epitopes can detect changes in conformation.^31,32^ Time-of-flight secondary ion mass spectrometry (ToF-SIMS) provides information about the exposure of specific amino acid species within the top 1–2 nm of an adsorbed protein,^33^ which can demonstrate differences in orientation or even conformation for rare or asymmetrically distributed amino acid species.^26,27,30,34,35^ Near-edge x-ray absorption fine structure (NEXAFS) can give information about protein backbone ordering and orientation and thus adsorbed protein orientation.^34^ Several studies have suggested that surface chemistry can affect the structure and function of adsorbed multimeric VWF.^36,37^ However, the use of multimeric VWF in those studies prevented the full use of all the methods described here except the antibodies and also did not allow a distinction between whether surface chemistry affects VWF function by controlling VWF stretching, or by direct interaction with the A1 domain.

In the studies presented here, we focus on the isolated A1 domain of VWF. The isolated A1 domain is far less complex than the full protein, and the structure is known,^38^ allowing a multitechnique approach that includes XPS, ToF-SIMS and NEXAFS with monoclonal antibodies and functional studies, to provide a new understanding of how A1 interacts with surfaces. This includes a systematic characterization of the function and structure of the isolated A1 domain adsorbed onto glass, tissue culture polystyrene (TCPS), and polystyrene (PS) surfaces. Understanding A1 adsorption behavior on synthetic surfaces is critical for designing *in vitro* experiments to mimic the *in vivo* system. It could also be beneficial to material design for blood contacting biomaterials because influencing VWF adsorption could impact thrombosis on implanted devices.

## II. MATERIALS AND METHODS

### A. Adsorption of VWF and A1 to surfaces

Glass coverslips (8 mm, ProSciTech, Thuringowa, Australia) were cleaned by sequential sonication in dichloromethane, acetone, and methanol. PS and TCPS plates (Corning) were sonicated in water before use. A1 generously provided by Miguel Cruz of Baylor College of Medicine was produced in *Escherichia coli* and contained residues 1238-1472 of mature VWF with 12 residues at the N terminus from the expression vector (MRGSHHHHHHGS).^38^ For antibody and functional studies, control surfaces were also prepared by incubation with bovine serum albumin (BSA) at 500 *μ*g/ml.

### B. Preparation of platelets

Platelets were isolated from the blood of healthy donors, which had been drawn into acid citrate dextrose tubes. Platelets were separated by differential centrifugation in the presence of Apyrase and PGE-1 (to inhibit platelet activation) and resuspended in Hepes Tyrodes buffer containing 200 *μ*g/ml BSA.^39^

### C. Platelet adhesion in flow

Platelet adhesion studies were performed in a parallel plate flow chamber, using lower surfaces prepared as described above with 10 *μ*g/ml A1, and blocked with BSA (200 *μ*g/ml) overnight at 4 °C. A 300 *μ*l bolus of washed platelets was introduced into a Glycotech™ flow chamber and allowed to settle for 30 s, before phosphate buffered saline (PBS)-BSA buffer was pushed through the chamber at the indicated shear stress and platelet–surface interactions observed with a 10× objective and CCD camera. To test specificity of platelet-A1 interaction, platelets were incubated with the AK2 anti-GPIbα antibody^40^ (Abcam) at a concentration of 25 *μ*g/ml for 15 min at room temperature prior to use in these studies. To calculate the translocation velocity, time-lapse videos were taken with a 10× objective, at 1 frame per second, and the platelets in the videos tracked using SVCell RS (DRVision Technologies LLC, Bellevue WA). The tracks were analyzed using custom scripts to calculate the velocity of each platelet at each time point. If the velocity in any frame was equal to or greater than half the hydrodynamic velocity (1.5 *μ*m times the shear rate, or the estimated velocity of fluid at the midpoint of a platelet touching the surface), then the platelet was assumed to be unbound at that time point. The average velocity of all bound platelets was calculated at each time point, and this value averaged over all time points at a given shear stress to calculate the translocation velocity at a given shear stress.

### D. Antibody binding through enzyme-linked immunoassay

PS (Corning), TCPS (Corning) and glass-bottom (MatTek) 96-well plates were incubated with 10 *μ*g/ml A1 domain for 2 h at 37 °C, and blocked with 200 *μ*g/ml BSA at room temperature for 18 h. Wells were then incubated with the mouse monoclonal antibodies 6G1, CR1, 5D2 (generously provided by the Lopez lab at Bloodworks Northwest) for 1 h at 37 °C, washed with Tris buffered saline (150 mM NaCl, 10 mM Tris) containing 0.2 vol. % Tween 20, incubated with goat antimouse antibodies conjugated with horseradish peroxidase for 1 h at 37 °C and washed again.

### E. XPS

Substrates were allowed to equilibrate overnight with PBS (137 mM NaCl, 2.7 mM KCl, and 10 mM phosphate) *p*H 7.4 at room temperature, and then incubated in VWF A1 solutions at the desired concentrations for 2 h at 37 °C. Following adsorption, substrates were rinsed twice in stirred PBS buffer to remove loosely bound protein, then three times in stirred water to remove buffer salts.^41^ Samples were dried in a nitrogen stream, and then were kept under an inert nitrogen atmosphere until analysis. XPS data were collected on a Surface Science Instruments S-Probe instrument with a monochromatized aluminum Kα x-ray source and electron flood gun for charge neutralization. Survey and detail scans were acquired at a 150 eV pass energy and a 55° takeoff angle, defined as the angle between the surface normal and the analyzer. For each concentration, two samples were analyzed and three spectra were collected on each sample. Spectra were analyzed using the Service Physics ESCA 2000A analysis software. Since nitrogen is unique to the adsorbed protein and not present in the substrates, nitrogen signal can be related to adsorbed protein amount.^21,42–44^

### F. ToF-SIMS

Samples were prepared as for XPS analysis. Data were acquired on an ION-TOF 5-100 instrument (IONTOF GmgH, Munster, Germany) using a Bi_3_^+^ primary ion source under static conditions (primary ion dose <10^12^ ions/cm^2^). Spectra were obtained from 100 × 100 *μ*m areas and five positive ion spectra were collected from each sample. The Bi_3_^+^ ion current ranged from 0.15 to 0.35 pA. A low-energy electron beam was used for charge compensation. Mass resolution (m/Δm) of the positive ion spectra was typically between 5500 and 7000 for the m/z = 27 peak. Prior to analysis, spectra were mass calibrated to the CH_3_^+^, C_2_H_3_^+^, C_3_H_5_^+^, and C_7_H_7_^+^ peaks. If the C_7_H_7_^+^ signal saturated the detector (on bare PS substrates), C_8_H_7_^+^ was used for calibration instead of C_7_H_7_^+^. Peaks were identified that corresponded to amino acid peaks. Amino acid peaks that overlapped with substrate peaks were then eliminated from analysis by identifying peaks with a normalized intensity that was greater than 15% of the sum of all amino acid peak intensities. Peaks used for analysis are listed in supplementary Table I,^45^ along with the corresponding amino acid.^41^ Intensity of amino acid peaks of interest (Trp and Cys) were normalized to the sum of all amino acid peaks to account for variations in protein surface concentration.

### G. NEXAFS

Samples were prepared as for XPS analysis. NEXAFS spectra were acquired at the National Synchrotron Light Source U7A beamline at Brookhaven National Laboratory using an elliptically polarized beam with ∼85% p polarization. This beamline uses a monochromator with a 600 L/mm grating that provides a full-width half-max resolution of ∼0.15 eV at the carbon K-edge (285 eV). The monochromator energy scale was calibrated using the 285.35 eV C 1 s−π* transition from a graphite transmission grid placed in the x-ray path. The partial electron yield for the nitrogen K-edge spectrum was monitored by a detector with the bias voltage maintained at −360 V. The signal was divided by the beam flux during data acquisition. Samples were mounted to allow rotation about the vertical axis to alter the angle between the incident x-ray beam and the sample surface. Data were collected at different NEXAFS angles, defined as the angle between the incident x-ray beam and the sample surface.

## III. RESULTS

### A. A1 adsorbed onto PS, TCPS, and glass surfaces are functionally different

**Fig. 1.**
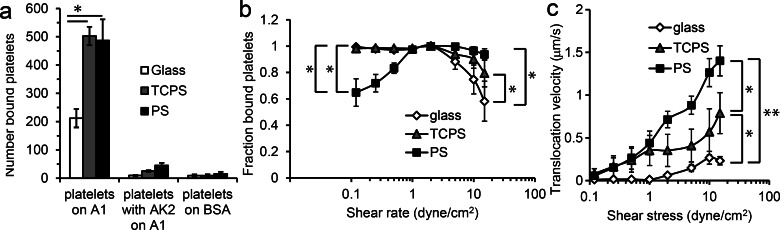
Platelet adhesion on adsorbed A1. In all panels, 10 *μ*g/ml A1 was adsorbed onto the indicated surfaces. (a) Number of platelets binding at 2 dyne/cm^2^ for the indicated conditions. Mean ± SD, n = 2. (b) After platelets bound to A1, flow was turned up or down every 30 s and the fraction of platelets remaining bound was counted at each shear stress. Mean ± SD, n = 5. (c) In the same experiment, the velocity of the bound platelets at each shear stress was calculated by tracking the individual platelets. Mean ± SD, n = 5. In all panels, a *p* <0.05 is indicated by * and a *p* <0.001 by **, for the unpaired one-sided Students' *t*-test between the two data points indicated.

To test for functional differences between A1 adsorbed onto different surfaces, we measured binding of platelets to the various surfaces in flow. After platelets were allowed to settle onto the surface, unbound platelets were washed away at a shear stress of 2 dyne/cm^2^ and the number of bound platelets counted. While comparable numbers of platelets adhered to A1 adsorbed onto PS and TCPS surfaces, fewer platelets adhered to A1 adsorbed onto glass surfaces. [Fig. 1(a)]. On all surfaces, this binding was mediated by the platelet receptor GPIbα, because binding was strongly inhibited when GPIbα was first blocked with the monoclonal antibody AK2 [Fig. 1(a)]. Moreover, the platelets were binding specifically to the adsorbed A1 domain rather than something else on the BSA-blocked surface, since almost no platelets bound to surfaces with adsorbed BSA [Fig. 1(a)].

To examine the ability of platelets to bind to A1 on the three surfaces at high shear stress, we turned the flow up stepwise from 2 to 15 dyne/cm^2^. The fraction of bound platelets was calculated relative to the initial number of platelets on the surface at 2 dyne/cm^2^. When shear stress was increased, platelets detached to some degree from all three surfaces, but significantly more platelets detached from the glass surface [Fig. 1(b)]. We then repeated this process, but this time turned the flow down stepwise from 2 to 0.1 dyne/cm^2^ instead of up. When shear stress was decreased, only the platelets bound to A1 on the PS surface detached significantly [Fig. 1(b)], so that platelets binding to PS surfaces demonstrated shear-enhanced adhesion, in which they detach below a threshold shear stress. Individual platelets were then tracked in both sets of videos to calculate translocation velocities, because slower translocation is one measure of increased binding strength. At all but the lowest shear stresses, the bound platelets translocated most quickly across A1 on PS surfaces and most slowly across A1 on glass surfaces [Fig. 1(c)]. No morphological changes were observed in platelets in any of these conditions, suggesting that the presence of activation inhibitors (see Subsection II B) prevented platelet activation as intended.

Together, these data demonstrate that A1 adsorbed onto PS mediates stronger adhesion than A1 adsorbed onto glass in some ways (more initial attachment and less detachment at high flow), but weaker adhesion in other ways (increased detachment at low flow and faster translocation). Instead of using the strength of adhesion as a measure of function, it may be more useful to consider the characteristics of adhesion. When VWF is immobilized on the endothelium *in vivo*, platelets bind only above a shear threshold and translocate rapidly across the surface.^46^ However, various structural changes in A1 have been shown to reduce platelet translocation velocities and increase the number of platelets binding at low flow, sometimes even while decreasing the number binding at high flow.^16,18^ Therefore, the biological function of A1 adsorbed onto PS is typical of native VWF, while the biological function of A1 adsorbed onto TCPS and especially on glass is more typical of various alternate A1 structures that retain ability to bind specifically to platelet GPIbα, but with altered characteristics of binding.

### B. XPS: Similar amounts of A1 are adsorbed onto the different surfaces

**Fig. 2.**
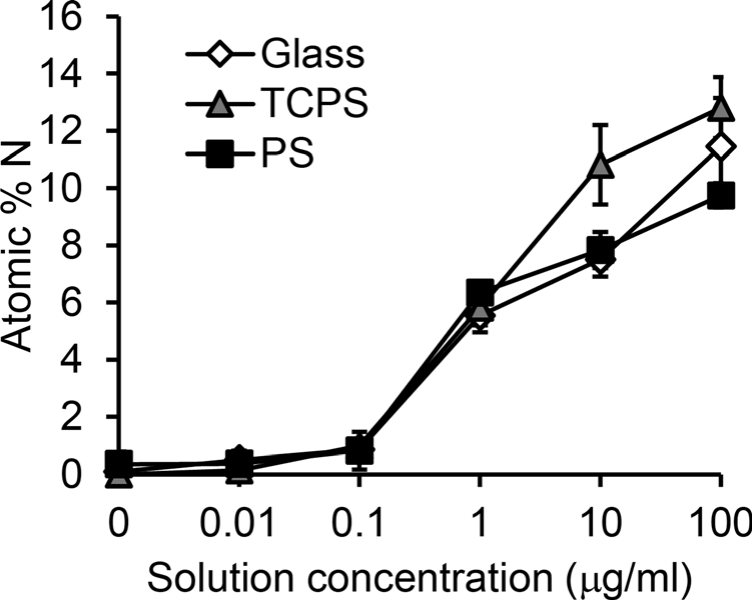
XPS results for A1 adsorbed onto glass, TCPS, and PS surfaces. XPS shows comparable amounts of nitrogen, and therefore protein, on each surface over the measured concentration range. Mean ± SD, n = 6.

One possible explanation for at least some of the differences in GPIbα binding of absorbed A1 could be differences in the site density, or coverage, of A1 on the various surfaces.^47^ For all three surfaces, nitrogen is unique to adsorbed proteins. Therefore, the XPS nitrogen signal can be used to track the amount of protein on each surface.^21,43,48^ The full XPS determined elemental compositions for all samples are listed in supplementary Table II.^45^ On all surfaces, the nitrogen percentage increased as the solution concentration increased, reaching 10–13 atomic % nitrogen for A1 absorbed from 100 *μ*g/ml solutions (Fig. 2). For A1 adsorption from each solution concentration, with the exception the 10 *μ*g/ml solution, there is no significant differences in the detected XPS nitrogen percentages on the three different surfaces. For adsorption from the 10 *μ*g/ml solution, a slightly higher XPS nitrogen percentage is observed on the TCPS surface compared to the glass and PS surfaces. The nitrogen atomic percentage in A1 is 17%, as calculated from the amino acid structure, so the approach of the measured XPS atomic % nitrogen to this value is consistent with the formation of approximately a monolayer of A1 on the surfaces since the dimensions of A1 [approx. 3.5 × 5 × 5 nm (Ref. 38)] are similar to the 5 nm sampling depth of XPS for a photoelectron take-off angle of 55°.

These results do not support the hypothesis that differences in platelet adhesion are due to differences in A1 surface coverage. In particular, for adsorption from 10 *μ*g/ml solutions, the amount of adsorbed A1 was comparable on the glass and PS surfaces, but these surfaces showed significantly different biological behavior.

### C. Antibodies: A1 binds with different orientation and/or conformation

To investigate the orientation or conformation of adsorbed A1 on the different surfaces, we examined the binding of three previously characterized monoclonal antibodies to A1 using enzyme-linked immunoassay (ELISA). 6G1 recognizes a linear conformation independent epitope at the C terminus of the A1 domain.^17^ CR1 and 5D2 both recognize unknown, nonlinear conformation-sensitive epitopes,^17,40^ and inhibit VWF binding to platelets, but it is unknown if they mask the GPIbα-binding site in A1 or stabilize an inactive conformation.

**Fig. 3.**
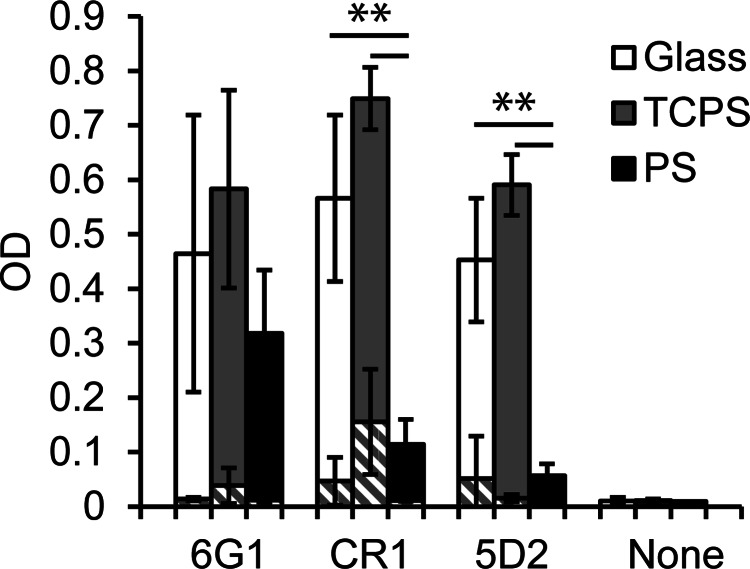
Differential recognition of A1 adsorbed from 10 *μ*g/ml solutions onto glass, TCPS, and PS surfaces by 6G1, CR1, and 5D2 monoclonal antibodies. The conformation-independent antibody 6G1 shows approximately the same level of binding, while the conformation-sensitive antibodies CR1 and 5D2 show reduced binding to A1 bound to PS. Negative controls of antibody binding to BSA surfaces (striped bars) and binding of the secondary antibody with no primary antibody showed minimal activity. Mean ± SD, n = 6–9. A *p* <0.001 is indicated by **, for the unpaired one-sided Students' *t*-test between the two data points indicated.

When A1 was adsorbed onto glass, TCPS, and PS surfaces, antibody 6G1 showed no statistically significant difference in binding (p > 0.05) among the three surfaces (Fig. 3). This is consistent with the prior observation that 6G1 is not conformation dependent, and suggests a comparable A1 concentration on each surface, consistent with the XPS results. In contrast, antibodies CR1 and 5D2 demonstrated reduced binding to A1 adsorbed onto PS relative to A1 on the other two surfaces (p < 0.05) (Fig. 3). This result could be consistent with a different orientation or conformation of A1 on PS compared to that on glass or TCPS. Previous characterizations of CR1 and 5D2 provide insufficient information about the location or conformation of the epitopes to provide more specific information about the structure of adsorbed A1.

### D. ToF-SIMS shows differential exposure of amino acid side chains

Differences in orientation and conformation of A1 adsorbed onto the three surfaces were further explored by ToF-SIMS. Due to their asymmetric distribution within A1 and importance in protein function, ToF-SIMS experiments were focused on the exposure of cysteine and tryptophan. A1 contains one Trp (Trp550) that electrostatically interacts with GPIbα during binding,^8^ so Trp exposure gives information about the accessibility to part of the large GPIbα binding site. A1 also contains two Cys residues that form a functionally important disulfide bond^18,49^ located distal to the GPIbα-binding site.

**Fig. 4.**
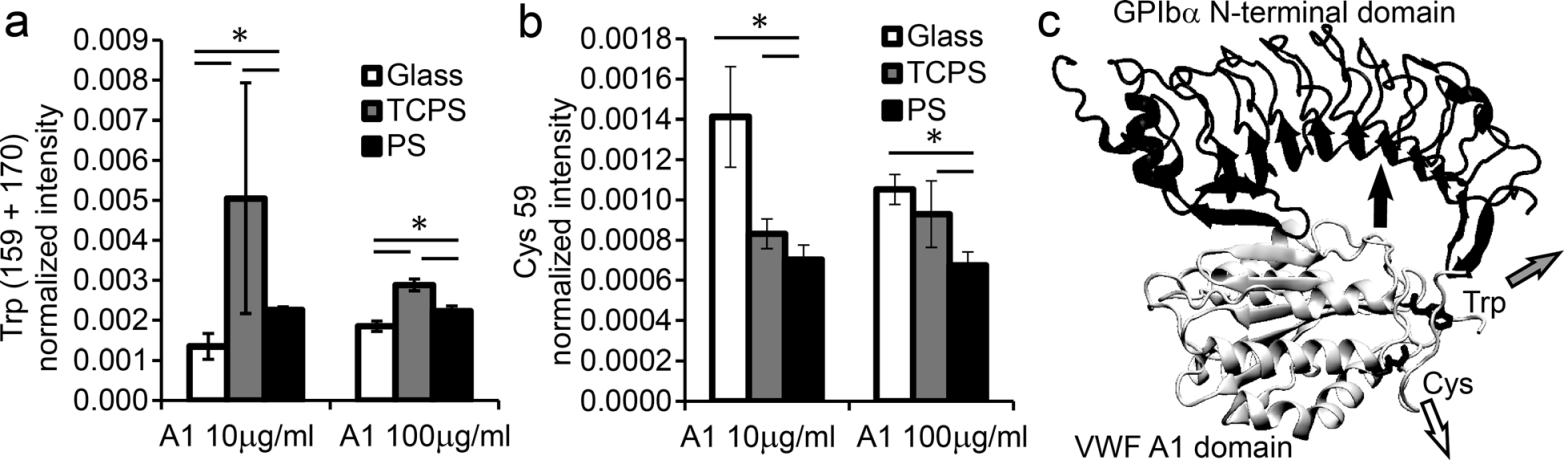
ToF-SIMS peak intensity of (a) Trp peaks (m/z 159 + 170) and (b) Cys peak (m/z 59) normalized to the sum of the amino acid peaks. A1 adsorbed onto glass, TCPS, and PS surfaces from 10 and 100 *μ*g/ml solutions. (a) Trp exposure was lowest when A1 was adsorbed onto the glass surface. (b) Cys exposure was lowest when A1 was adsorbed onto PS surfaces and highest when A1 was adsorbed onto glass surfaces. Mean ± SD, n = 10. A *p* <0.05 is indicated by *, for the unpaired one-sided Students' *t*-test between the two data points indicated. (c) The crystal structure of A1 bound to the N-terminal domain of GPIbα [PDB 1U0N (Ref. 62)] with Trp and Cys residues highlighted.

Trp solution exposure was examined by taking the ratio of the combined Trp m/z 159 + 170 peak intensities to the sum of the intensities of all amino acid peaks. Cys solution exposure was examined by taking the ratio of the Cys m/z 59 peak intensity to the sum of the intensities of all amino acid peaks. For each A1 adsorption concentration, the Trp exposure was lower on the glass surface than on the PS surface [Fig. 4(a)], while the Cys exposure was higher on the glass surface than on the PS surface [Fig. 4(b)]. The exposure of Cys for A1 adsorbed onto TCPS was intermediate between that of A1 on glass and PS surfaces, but Trp appeared to be more exposed on the TCPS surface compared to either the glass or PS surfaces. However, the Trp signal was especially inconsistent (i.e., high standard deviation) for A1 adsorbed onto TCPS surfaces from 10 *μ*g/ml solutions.

The observed differences in Trp and Cys exposures on the three surfaces are consistent with different orientations of A1 on these three surfaces, in which the Trp-containing GPIbα-binding site is preferentially faced away from the PS surface [as illustrated by the black arrow in Fig. 4(c)], the Trp region of the GPIbα-binding site is preferentially faced away from the TCPS surface [gray arrow in Fig. 4(c)] and the disulfide bond is preferentially faced away from the glass surface [white arrow in Fig. 4(c)]. This difference in orientation would predict that platelet adhesion would be strongest when A1 is adsorbed onto PS, intermediate when A1 is adsorbed onto TCPS, and weakest when A1 is adsorbed onto glass. Therefore, the preferential orientations described above could explain the differences in initial adhesion and in ability to remain bound at high shear stress. However, differential orientation could not fully explain all the biological data (e.g., detachment of platelets at low shear stress from A1 adsorbed onto PS surfaces), because orientation will only affect the amount of exposed GPIbα-binding sites, which would not have different effects at high versus low shear stress.

An alternative explanation of the differences in Cys exposure is that the three surfaces stabilize different conformations of the A1 domain, which could result in different degrees of reduction of the disulfide bonds or Cys exposure. If this is the case, then it would indicate that glass and TCPS surfaces stabilize an alternative conformation of the A1 domain where the disulfide bond is reduced and/or more exposed. Indeed, Cys exposure (glass > TCPS > PS) is anticorrelated with normal biological function (PS > TCPS > glass), supporting the notion that Cys exposure reflects stabilization of an alternative conformation that although functional to binding GPIbα, has altered binding properties.

Comparing respective Cys and Trp exposure on different surfaces, we observed less differences in the ToF-SIMS Cys and Trp intensities when A1 is adsorbed from higher concentration versus lower concentration solutions (Fig. 4). This further suggests that conformation plays a role in observed biological functions. Adsorption from higher solution concentrations results in higher A1 surface densities, which could inhibit conformational changes (i.e., less open surface area for A1 to denature and spread out over), resulting in more similar behavior among the different surfaces.

In summary, the ToF-SIMS data point to a difference in orientation when A1 is adsorbed onto the three surfaces, or to differential stabilization of alternative conformations, or both. However, there are no possible orientational differences that correlate exposure of the GPIbα-binding site with strength of adhesion in all measurements, while there are possible conformational differences that can explain both Cys exposure and biological differences. To explain Trp and Cys ToF-SIMS data and biological data, the most likely explanation is that both conformation and orientation are different on the different surfaces.

### E. NEXAFS shows differences in amide backbone ordering

**Fig. 5.**
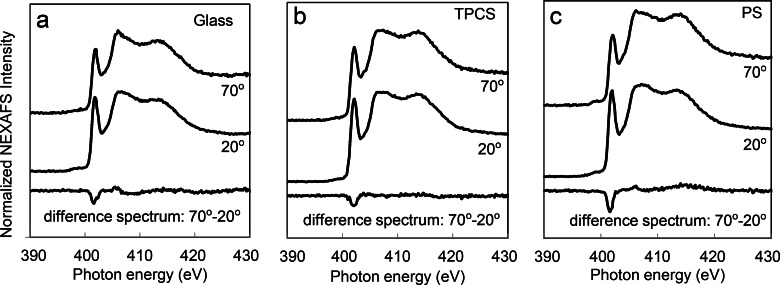
NEXAFS nitrogen K-edge spectra of A1 adsorbed onto (a) glass, (b) TCPS, (c) PS surfaces. Spectra collected at incident angles of 70° and 20°. Strongest dichroism of the amide π* feature was observed when A1 was adsorbed onto a PS surface, as shown in the difference spectrum of 70°–20°. A1 was adsorbed from 10 *μ*g/ml solutions. The spectra are offset for clarity (Fig. 1). Current–voltage characteristics of diodes made from films grown at three different temperatures.

In addition to examining the side chain exposure, we were also interested in the protein backbone secondary structure. The NEXAFS nitrogen K-edge spectra of A1 adsorbed onto glass, TCPS, and PS surfaces exhibit strong π* absorption resonances around 400 eV (Fig. 5) corresponding to the protein amide backbone.^50–52^

In protein films containing ordered structures, the intensity of the π* feature varies with changing NEXAFS angle.^34^ Because the amide π* orbitals within α-helices are oriented in many directions, they typically only contribute slightly to the angle-dependent NEXAFS signal.^53^ In contrast, ordered β-sheet structures typically contribute the majority of the angle-dependent NEXAFS signal for surface bound peptides and proteins, as shown in previous studies examining the orientation peptides and proteins on surfaces.^34,54^

To examine the NEXAFS angle dependence, we calculated the difference spectrum between spectra collected at 70° and 20° incident angles (Fig. 5). The difference spectra exhibited some dichroism when A1 was adsorbed onto all three surfaces. A1 adsorbed onto a PS surface shows a slightly stronger dichroism, suggesting that the amide π* orbitals are relatively more aligned on this surface compared to the other two surfaces. This could be due to A1 adopting a wider range of orientations or a more disordered or dynamic conformation on the glass and TCPS surfaces. The dichroism is somewhat weak on all the surfaces, likely because the beta sheets within A1 are not completely parallel, and the NEXAFS signal is averaged over all the amide π* orbitals in A1.

The negative polarity of the dichroisms shows the x-rays are more strongly coupled with the amide π* orbitals when the x-rays are at a glancing incident angle compared to a near-normal incident angle. This suggests that the amide bonds are, on average, oriented more parallel to the surface than perpendicular to the surface on all three surfaces.^34^

In summary, the NEXAFS data demonstrate that the beta sheets in A1 maintains some degree of structure and are oriented more parallel to the surface when A1 is adsorbed onto all three surfaces, but that beta sheet structure and/or parallel orientation are slightly higher for A1 adsorbed onto PS surfaces. These data are also consistent with the differences in preferential orientation illustrated in Fig. 4(c), but would be consistent with other orientations as well.

## IV. DISCUSSION

The results from this study demonstrated that adsorption of A1 onto glass, TCPS and PS surfaces affects its biological function differently, with the greatest differences observed between the function of A1 adsorbed onto a glass surface versus onto a PS surface. Specifically, A1 adsorbed onto PS surfaces mediated the strongest adhesion in some ways but the weakest adhesion in other ways, resulting in platelet adhesion that was most characteristic of the shear-enhanced translocation of platelets bound to plasma VWF *in vivo*. In contrast, A1 adsorbed onto glass exhibited a biological function that is most characteristic of various activated A1 structures; that is, platelets bound in a shear-inhibited manner with little translocation. A1 adsorbed onto TCPS exhibited an intermediate biological function. None of these differences can be caused by a difference in surface concentration because both XPS (Fig. 2) and conformation-independent antibodies (Fig. 3) detected no significant differences in the amount of adsorbed A1 on the three surfaces under these conditions. Some of the biological functional differences could be explained by the preferential orientations of A1 on the three surfaces such as that illustrated in Fig. 4(c), which are consistent with the relative exposure of Trp and Cys determined by ToF-SIMS and with the polarization dependence measured by NEXAFS. However, no difference in orientation can simultaneously explain why platelets adhere better in some ways and worse in others when comparing A1 adsorbed onto two surfaces, because orientation should just affect the amount of functional GPIbα binding site. Differential stabilization of native functional and denatured nonfunctional conformations of A1 also fails to explain these simultaneous differences because denaturation of A1 would also only affect the amount of functional GPIbα binding sites. Because our data cannot be completely explained by differences in concentration, orientation, or even denaturation, our study demonstrates that at least some of the functional differences must reflect different conformations of A1 with different biological functions.

A1 is known to take on an alternative conformation in some conditions that is more active^49,55^ than the native conformation. This “activated” conformation is normally stabilized by tensile force on the A1-GPIbα bond; A1 mediates shear enhanced adhesion of platelets,^16,56^ and mediates formation of force-activated catch bonds with GPIbα.^16^ The activated conformation can also be stabilized by von Willebrand disease type 2B mutations, because A1 containing such mutations mediates shear-inhibited adhesion of platelets with a slower translocation velocity^16,56^ and mediates force-inhibited slip bonds with GPIbα.^16^ Reduction of the disulfide bond in the A1 domain has also been associated with shear-inhibited adhesion and a slower translocation velocity.^18^ Finally, shortening of the N-terminal flanking region of A1, which covers the disulfide bond in crystal structures, is also known to stabilize the activated conformation^57,58^ and cause force to inhibit rather than activate A1.^59^ Therefore, a wide range of structural changes in A1 can create the same biological function—shear-inhibited adhesion with slower translocation velocities—as does adsorption of A1 onto glass. In contrast, when A1 domain is adsorbed onto collagen, it mediates shear-activated adhesion and force-activated catch bonds^60^ similar to A1 adsorbed onto PS.^16^ Remarkably, the CR1 and 5D2 antibodies fail to recognize A1 adsorbed onto collagen^61^ and PS (Fig. 3) but not onto glass or TCPS (Fig. 3). Together, these observations strongly support the conclusion that glass and TCPS stabilize an activated conformation of the A1 domain that is distinct from the native conformation that is stabilized by PS and collagen.

The exact structure of the activated conformation of A1 remains unknown, because all of the crystal structures of A1 in isolation^38,62^ or bound to GPIb,^8,9,63^ appear nearly identical with only minor transient differences.^64^ Remarkably, reduction of the disulfide bond and shortening of the N-terminal flanking region could both be associated with the increased Cys exposure as well as with activation of A1. Because Cys exposure in our studies correlated with slower and shear-inhibited translocation similar to that of activated forms of A1, it is possible that glass and TCPS reduce the disulfide bond or dislodge the N-terminal flanking region to cause activation of A1. However, as noted above, the increased Cys exposure could also be explained by changes in orientation. Moreover, the surfaces may affect conformation through another mechanism such as conformational control of the regulatory region that is characterized by the activating type 2B mutations.^65^ Therefore, the nature by which some surfaces appear to stabilize the active conformation of the A1 domain remains to be determined.

It is worth comparing our studies to previous studies that measured the effect of shear on platelet adhesion to surface-adsorbed VWF. When wild type A1 or plasma VWF was adsorbed onto PS surfaces,^16,56^ platelets bound in a shear-enhanced manner, consistent with our studies. Shear-inhibited adhesion was sometimes observed when A1 (Ref. 19) or plasma VWF (Ref. 46) was adsorbed onto glass surfaces, consistent with our findings. In other cases,^12,66^ shear enhanced adhesion was observed, but these studies measured adhesion in the presence of red blood cells, which push platelets into the wall at high shear stress, providing an alternative possible mechanism for shear-enhanced adhesion that complicates interpretation. Further studies are needed to address what kinds of surfaces can activate A1, and whether activation is still significant when higher concentrations of A1, plasma VWF, or mixtures of proteins are adsorbed onto surfaces. It also remains to be determined how activation or even partial activation of VWF might affect thrombosis and healing *in vivo*. Because small numbers of activated domains may have much more profound biological effects than the loss of small numbers of domains to poor orientation or denaturation, these questions are important to answer for the design of biomaterials that contact the blood.

Von Willebrand Factor is not the only blood protein in which the native conformation is inactive relative to an activated conformation. These proteins may be referred to as autoinhibited, and include the majority of soluble proteins in the blood that are involved in cell adhesion or clotting. For example, fibrinogen in the soluble form makes up a large fraction of blood proteins, where it does not bind blood cells. However, these proteins bind platelets and immune cells when adsorbed to surfaces,^12,67^ possibly due to conformational changes that mimic natural polymerization processes.^67^ Fibronectin similarly binds to cell adhesion receptors in a conformation-dependent manner that is controlled by surface adsorption.^68^ Clotting factors are also autoinhibited and while most are activated by cleavage, the intrinsic, or contact, pathway is initiated by binding of a complex of clotting factors to exposed collagen, but is also activated by contact of blood with surfaces.^3^ In most cases, the structural basis of autoinhibition and activation are not clear, and the structural changes that occur upon surface adsorption are almost never known.

Most critically, there remains much research to be done to determine the details of how blood proteins are activated by surface adsorption and how this process depends on the chemical properties of the surface. This information is needed to provide guidance about designing biomaterials that stabilize the native rather than activated forms of key blood proteins. Ratner has discussed the history and challenges associated with addressing this “blood compatibility catastrophe.”^69,70^ More recently, an assessment of the current state of blood–biomaterials interaction and what is needed to advance our understanding of these interactions was discussed at the “74th International IUVSTA Workshop on Blood-Biomaterial Interactions: Surface Analysis meets Blood Compatibility.”^71^ There are three aspects that need to be considered when addressing blood–biomaterials interactions. The first item is the design and engineering of biocompatible surfaces. While some progress toward biocompatibility has been made by using biomaterials with species such as poly(ethylene glycol) and zwitterions on their surface, we still have not developed a biomaterial that can match the performance of the native endothelial surfaces of the vascular system. The second item is detailed characterization of biomaterial surfaces and biomolecules such as proteins interacting with those surfaces. Significant progress has been made in the level of detail that can be obtained from biomedical surface analysis studies, but we still have yet to achieve an atomic level structure for surface bound proteins. For surface bound proteins, it is essential to not just determine the amount of protein present, but its structural details such as orientation and conformation. The third item is how to develop meaningful test of blood interactions with biomaterials. The complex, multicomponent and highly interactive nature of blood makes this a particularly difficult challenge. However, addressing all three of these issues is required to advance our understanding of blood–biomaterial interactions. The results from the current study, which combines three different biomaterial surfaces, surface analysis of A1 adsorbed onto those surfaces, and biological measurements of platelet activity, represent a step forward in this process.

## V. CONCLUSIONS

These studies demonstrate that the A1 domain of VWF has fundamentally different biological activity when adsorbed onto different surfaces. Platelets translocate rapidly on A1 adsorbed onto PS surfaces, and demonstrate shear-enhanced adhesion in that they detach at low rather than high shear stress. In contrast, platelets translocate more slowly on A1 adsorbed onto TCPS surfaces and are nearly stationary on A1 adsorbed onto glass surfaces, and demonstrate shear-inhibited adhesion in that they detach at high but not low shear stress. XPS and antibodies detected no significant difference in the amount of A1 adsorbed onto the three surfaces. While ToF-SIMS and NEXAFS data suggest that A1 may be oriented differently on the different surfaces, no difference in orientation can explain why platelets would detach at low flow from some surfaces and at high flow from others, so the differences in biological function must be caused by conformational differences. Indeed, both ToF-SIMS Cys exposure and conformation-sensitive antibody binding suggest that A1 retains its native conformation when adsorbed onto PS surfaces, while TCPS surfaces and especially glass surfaces stabilized an alternative activated conformation of A1 that likely resembles the activated form of A1 that is also stabilized by disease-causing mutations. Regardless of the specific structure of the activated forms of A1, these studies demonstrate that it is not enough to determine the amount of various proteins that bind to different biomaterials placed in contact with the blood; instead, it is necessary to understand how different surfaces control the conformation of the many blood proteins that are capable of undergoing activating conformational changes.
